# Expression of concern: ‘Chimpanzee vowel-like sounds and voice quality suggest formant space expansion through the hominoid lineage’ (2022) by Grawunder *et al.*

**DOI:** 10.1098/rstb.2022.0476

**Published:** 2023-02-13

**Authors:** Sven Grawunder, Natalie Uomini, Liran Samuni, Tatiana Bortolato, Cédric Girard-Buttoz, Roman M. Wittig, Catherine Crockford

Following the publication of ‘Chimpanzee vowel-like sounds and voice quality suggest formant space expansion through the hominoid lineage’ *Phil. Trans. R. Soc. B* **377**: 20200455 (Published online: 15 November 2021), it has been brought to the attention of the Editorial team that there are concerns regarding this study.

An investigation into these aspects is under way, and the journal is, therefore, issuing an expression of concern and will notify readers as to the results of our investigation as soon as possible.

